# Nitrogen Removal Capacity of Microbial Communities Developing in Compost- and Woodchip-Based Multipurpose Reactive Barriers for Aquifer Recharge With Wastewater

**DOI:** 10.3389/fmicb.2022.877990

**Published:** 2022-05-24

**Authors:** Maria Hellman, Cristina Valhondo, Lurdes Martínez-Landa, Jesús Carrera, Jaanis Juhanson, Sara Hallin

**Affiliations:** ^1^Department of Forest Mycology and Plant Pathology, Swedish University of Agricultural Sciences, Uppsala, Sweden; ^2^Institute of Environmental Assessment and Water Research (IDAEA), CSIC, Barcelona, Spain; ^3^Associate Unit, Hydrogeology Group (UPC-CSIC), Barcelona, Spain; ^4^Department of Civil and Environmental Engineering, Universitat Politècnica de Catalunya (UPC), Barcelona, Spain

**Keywords:** water reuse, wastewater, nature-based solutions, soil aquifer treatment, reactive barriers, nitrogen removal, N-transforming microorganisms

## Abstract

Global water supplies are threatened by climate changes and the expansion of urban areas, which have led to an increasing interest in nature-based solutions for water reuse and reclamation. Reclaimed water is a possible resource for recharging aquifers, and the addition of an organic reactive barrier has been proposed to improve the removal of pollutants. There has been a large focus on organic pollutants, but less is known about multifunctional barriers, that is, how barriers also remove nutrients that threaten groundwater ecosystems. Herein, we investigated how compost- and woodchip-based barriers affect nitrogen (N) removal in a pilot soil aquifer treatment facility designed for removing nutrients and recalcitrant compounds by investigating the composition of microbial communities and their capacity for N transformations. Secondary-treated, ammonium-rich wastewater was infiltrated through the barriers, and the changes in the concentration of ammonium, nitrate, and dissolved organic carbon (DOC) were measured after passage through the barrier during 1 year of operation. The development and composition of the microbial community in the barriers were examined, and potential N-transforming processes in the barriers were quantified by determining the abundance of key functional genes using quantitative PCR. Only one barrier, based on compost, significantly decreased the ammonium concentration in the infiltrated water. However, the reduction of reactive N in the barriers was moderate (between 21 and 37%), and there were no differences between the barrier types. All the barriers were after 1 year dominated by members of Alphaproteobacteria, Gammaproteobacteria, and Actinobacteria, although the community composition differed between the barriers. Bacterial classes belonging to the phylum Chloroflexi showed an increased relative abundance in the compost-based barriers. In contrast to the increased genetic potential for nitrification in the compost-based barriers, the woodchip-based barrier demonstrated higher genetic potentials for denitrification, nitrous oxide reduction, and dissimilatory reduction of nitrate to ammonium. The barriers have previously been shown to display a high capacity to degrade recalcitrant pollutants, but in this study, we show that most barriers performed poorly in terms of N removal and those based on compost also leaked DOC, highlighting the difficulties in designing barriers that satisfactorily meet several purposes.

## Introduction

Climate change with altered precipitation patterns, an increasing human population and expansion of urban areas threaten sustainable drinking water supplies globally (Wheeler and von Braun, 2013). A continuous decline in the long-term groundwater level is becoming increasingly problematic due to over-drafting, and freshwater supplies can be limited in densely urbanized areas. More than 50% of the world’s population lives in urban areas, and by 2050 it is expected to increase to 68% (United Nations [UN], 2018). Thus, there are increasing demands for transforming urban water solutions, including water reuse and reclamation, to close the urban water cycle (Valhondo and Carrera, 2019). Nature-based solutions are gaining interest, and one option is the application of managed aquifer recharge (MAR), a low-cost technology that is used for increasing groundwater resources and enhancing recharged water quality (Bekele et al., 2018; Silver et al., 2018). The effluent coming from wastewater treatment plants is a possible water resource for this purpose (Page et al., 2018). However, treated wastewater contains various contaminants, such as pharmaceuticals, personal care products, pesticides, pathogens, and nutrients, that have not been eliminated in the wastewater treatment plant (Loos et al., 2013; Petrie et al., 2015) and which affect the water quality and threaten the integrity of groundwater ecosystems.

The implementation of reactive barriers in MAR systems has been proposed to increase the removal of contaminants (Valhondo et al., 2014). A reactive barrier is a layer of carbon-rich material, for example, compost or woodchips, through which water percolates. This offers a range of redox conditions and adds sorption sites that facilitate biodegradation and retard the transport of pollutants through the system (Valhondo et al., 2018), but the efficiency and capacity to remove environmentally harmful pollutants are uncertain and will depend on the type of barrier, pollutant (Modrzyński et al., 2021), and the operation of the system. The addition of reactive barriers has shown promising results for the removal of organic micropollutants and for reducing the number of pathogens in the treated water (Schaffer et al., 2015; Valhondo et al., 2020a; Modrzyński et al., 2021). With respect to nitrogen (N) removal, reactive barriers can also support denitrification for the removal of nitrate ions (Beganskas et al., 2018; Grau-Martínez et al., 2018). However, the fate of N when ammonium-rich treated sewage water is used with compost as a reactive layer has not yet been well addressed and may even counteract the desired N removal (Modrzyński et al., 2021). Compost might add ammonium to the system by the mineralization of N-containing organic matter in the compost. Further, the introduction of compost will supply carbon (C) for co-metabolic degradation of pollutants, but could also increase the C to N ratio, which may further promote dissimilatory nitrate reduction to ammonium (DNRA), thereby retaining N in the system (Putz et al., 2018). Overall, the effects of compost addition on N removal are unclear and open the question regarding which microbial communities develop when ammonium-rich water percolates through the barrier.

Our aim was to evaluate compost- and woodchip-based reactive barriers for their ability to remove N when infiltrated with ammonium-rich treated sewage water and determine the microbial communities developing in these barriers, including the pathways underpinning N-transforming processes. We used a pilot facility representing the MAR technique soil aquifer treatment (SAT) with infiltration basins supplemented with compost- and woodchip-based barriers and an infiltration basin with only a sand filter (control) (Valhondo et al., 2020b). The basins were operated under alternating dry and wet cycles (Bouwer et al., 2008; Sharma and Kennedy, 2017), but with very long wet periods (Supplementary Figure 2). Nitrogen removal was studied together with the diversity and composition of the microbial communities developing during the first year of operation. The prevalence of the microbial N-transformation pathways nitrification, denitrification, nitrous oxide reduction, and DNRA was estimated by quantifying the abundance of key genes involved in N-transformation processes. We hypothesized that (1) the microbial communities developing in the barriers would affect the N-removal capacity by changing the genetic potentials for the various N-transforming processes, and (2) the compost-based barriers would develop the most diverse microbial communities, followed by the woodchip and the control barriers, because of the larger redox range offered in the compost-based barrier and the additional sorption sites for contaminants in both compost- and woodchip-based barriers.

## Materials and Methods

### System Description

**FIGURE 1:**
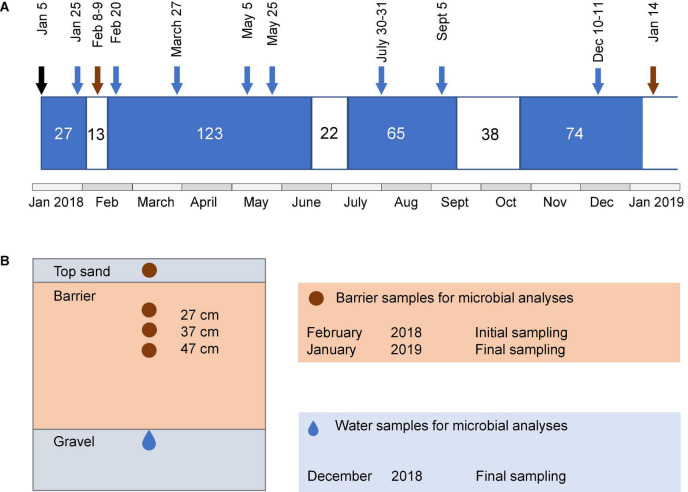
Schematic illustration of the operational periods of the system, sampling time points, and sampling locations. **(A)** Length of wet (blue) and dry periods (white) and time points for water and barrier sampling. Sampling of barrier samples is indicated by brown arrows and water samples after barrier passage are indicated with blue arrows. The black arrow indicates the date of the first wet cycle. Microbial community analysis in water samples was only performed for the last sampling occasion. **(B)** Cross-section of the side view of barrier compartment, showing regions from where samples for characterization of microbial communities were taken. Brown area indicates barrier and gray areas indicate sand and gravel.

The pilot aquifer recharge facility located at a wastewater treatment plant (WWTP) in the vicinity of Girona, Spain, has been described in detail previously (Valhondo et al., 2020b). The facility has reactive barriers constructed using compost- or woodchip-based materials for the infiltration of secondary effluent from the WWTP and a reference system with sand only. The compost used was based on garden waste, and the woodchips were from pine wood. The barriers were 110 cm deep, including a 10 cm top layer of fine sand, and the infiltration area was 3.6 m^2^. All except one of the barriers had vegetation with mixed plant species that were established naturally. In this study, we evaluated one woodchip-based barrier (WCB), three compost-based barriers varying in the proportions of sand, compost, clay, and presence of vegetation (CB1-, CB1, and CB2), and the reference (Ref) for their ability to reduce reactive N (ammonium and nitrate) and dissolved organic compounds (DOC) in the secondary-treated effluent water from the plant (Table 1). The purpose of clay was to provide sorption sites for cationic compounds. The concentrations of reactive N and DOC in the influent water to the barriers were determined on several occasions using the methods described in Valhondo et al. (2020b). The average influent ammonium (*n* = 31), nitrate (*n* = 29), and DOC (*n* = 12) concentrations were 57.4, 0.44, and 15.3 mg L^–1^, respectively (Supplementary Table 1 and Supplementary Figure 1). The oxygen level in the influent water was 3.76 mg L^–1^ (SD = 2.23, *n* = 28) and pH was 7.6 (SD = 0.22, *n* = 30; Supplementary Table 1). The pilot plant was operated in wet and dry cycles with four recharge periods of 27–123 days and three intermittent dry periods of 13–38 days (Figure 1A). The average inflow rate was 1 L min^–1^, corresponding to a recharge rate of 0.40 m day^–1^. To facilitate uniform infiltration, the influent was distributed through a 7 m long tubing, perforated every 20–25 cm, covering the recharge area.

**TABLE 1:** Barrier composition (% volume).

	CB1-	CB1	CB2	WCB	Ref
Vegetation	No	Yes	Yes	Yes	Yes
Sand	49	49	60	49	100
Compost	49	49	40	–	–
Woodchips	–	–	–	–	–
Clay	2	2	2	2	–

### Sampling of Water and Barrier Material

Water samples were taken from piezometers installed in a 20-cm thick layer of gravel under each of the reactive barriers (Figure 1B). Samples for chemical characterization were collected at eight time points during the wet periods (Figure 1A). Water for microbial analyses was collected in the middle of the last wet cycle, that is, after 45 wet days (10–11 December 2018). For this purpose, 80–150 ml of infiltrated water per barrier was filtered through a Sterivex^®^ polyethersulfone filter unit (0.22 μm pore size), and the filters were kept at −20°C until further analysis. In the following text, the term “water” refers to the water after passage through the barriers unless stated otherwise. Barrier material was collected during the dry periods by digging with a small spade 7 or 8 days after the first wet cycle (8–9 February 2018, initial sampling) and 6 days after the fourth wet cycle (14 January 2019, final sampling). Four depths were sampled: the top sand and at 27, 37, and 47 cm depth from the top of the upper sand layer (Figure 1B). During the last sampling, it was only possible to collect the top sand and the 27-cm layer of the reference, since the sand fell into the hole while digging. The samples were kept at −20°C until further analysis.

### Chemical Analyses of Water and Barrier Material and Calculation of N Removal

Dissolved organic carbon in the water was analyzed using a TOC-VCSH analyzer (Shimadzu, Kyoto, Japan) after filtration (0.22 μm) and acidification, ammonium (NH_4_^+^) concentration was analyzed using an ORION Ion Selective Electrode (ISE, Thermo Fisher Scientific, Waltham, MA, United States), and nitrate (NO_3_^–^) concentration was determined by ion chromatography as previously described (Valhondo et al., 2020b). The amount of reactive N removed (percent) was calculated per barrier and date (*n* = 1) as:

Nremoved=100×(total⁢Nin-total⁢Nout)/total⁢ Nin⁢where

total Nin=(NO3-+NH4+)ininflowwater(mgNL-)1and

total Nout=(NO3-+NH4+)inwaterafterbarrierpassage(mgNL)-1.

Dry weight was calculated by the amount of weight lost after drying overnight at 105°C. Organic matter content in the barrier samples was determined by weight lost after combustion. The pH of the barrier samples was measured in a sample: water slurry (1:2.5 weight/weight) after incubating for 1 h on a rotary shaker (200 rpm), followed by 1.5 h for the settling of solids in the dark.

### Molecular Methods

#### Sample Preparations and DNA Extraction

For the water samples, the filters were detached from the Sterivex^®^ cartridges and cut into halves. For each sample, one of the halves was cut into 8–10 pieces and transferred to a microcentrifuge tube. For the barrier material, approximately 0.4 g was used for the DNA extraction. The samples collected from the woodchip barrier were first sieved, and the size fractions > 2 mm were ground in a ball mill (Laarmann LMLW-320/2, Roermond, the Netherlands) using two steel balls (20 mm diameter) for maximum of six 10 second pulses at a frequency of 30 s^–1^. Before weighing the woodchip material for the extraction, the <2 mm and the milled > 2 mm fractions were thoroughly mixed in the same weight/weight ratio as before the sieving process. DNA was extracted from all samples using the Qiagen PowerSoil kit according to the manufacturer’s instructions, with a slight modification in the cell lysis step, where Precellys tissue homogenizer (set at 5,500 rpm for 30 s) was used. The extracted DNA was quantified using the Qubit fluorometer (Thermo Fisher Scientific, Waltham, MA, United States).

#### Quantitative PCR

To estimate the genetic potential for carrying out different N-transformation processes, the abundances of functional genes were determined by performing quantitative real-time PCR (qPCR) as described in Modrzyński et al. (2021), using 2 ng of template in each reaction. The abundances of *nirS* and *nirK* genes were used as proxies for the denitrifying community (Henry et al., 2004; Throbäck et al., 2004), *nosZ*I and *nosZ*II for the nitrous oxide-reducing community (Henry et al., 2006; Jones et al., 2013), *nrfA* for the DNRA performing community (Mohan et al., 2004; Welsh et al., 2014), and *amoA* (bacterial and archaeal) for the ammonia-oxidizing communities (Rotthauwe et al., 1997; Tourna et al., 2008). Primer sequences, reaction conditions, and thermal cycling protocols are summarized in Supplementary Table 2.

#### Sequencing and Bioinformatic Analyses of 16S rRNA Genes

To determine the diversity and composition of the microbial communities in the water and barrier samples, the V3–V4 region of the bacterial and archaeal 16S ribosomal RNA genes was amplified using the primer pair of pro341F and pro805R (Takahashi et al., 2014) as previously described (Hellman et al., 2019), with the slight modifications that the first PCR was performed in 25 μl of the reaction mixture and that purification steps were carried out using Sera-Mag™ magnetic beads (GE Healthcare, Chicago, IL, United States). Sequencing was performed on an Illumina^®^ MiSeq instrument using the 2 × 250 bp chemistry. The raw sequence dataset is available in the NCBI sequence read archive (SRA) under BioProject accession number PRJNA773712.

The sequences were processed as described in Hellman et al. (2019). Briefly, the trimmed and merged sequences were quality-filtered using a maximum expected error = 1, dereplicated using a minimum unique size = 2, clustered at 97% identity, and chimera-checked using VSEARCH (Rognes et al., 2016). Singleton operational taxonomic units (OTUs) were removed, and representative OTUs were classified using the non-redundant reference database SILVA version 132 (Yilmaz et al., 2014). A phylogenetic tree was constructed based on the aligned nucleotide sequences of representative OTUs using FastTree (Price et al., 2009) and the Jukes-Cantor and CAT model (Jukes and Cantor, 1969). Sequences representing chloroplasts and mitochondria were identified from the tree and removed. One sample (a barrier sample from CB2, February 2018) had only 94 sequences and was excluded from the analyses. The resulting dataset had 1,276,515 sequences, corresponding to 9,862 OTUs. The dataset was divided into separate sets for water and barrier samples (5 and 37 samples, respectively) and rarefied to the smallest sample size (20,364 counts per sample and 3,757 OTUs for water and 7,479 counts per sample and 7,634 OTUs for barrier). To partition OTUs identified as frequent members of the community from less frequent OTUs, a dispersion index (the ratio of the variance to the mean abundance, multiplied by the occurrence) was calculated for each OTU and used to model whether the OTUs followed a Poisson distribution. Theory predicts that rare OTU abundances are randomly (Poisson) distributed across sites and follow a log-series distribution, whereas frequently occurring OTU abundances are non-randomly distributed (Magurran and Henderson, 2003). OTUs occurring at a frequency above the 2.5% confidence limit of the Chi^2^ distribution were retained in the dataset (Saghaï et al., 2021). If not stated specifically, these frequently distributed communities are those analyzed and discussed in the following text. Analysis of the sequence data was carried out in R, version 4.0.3 (R Development Core Team, 2016).

### Statistical Analyses

“Phyloseq” (McMurdie and Holmes, 2013) was used to estimate Shannon entropy (H′). Pielou’s evenness index (J) was calculated from the Shannon entropy as J = H′/ln(S), where S is the total number of taxa in a sample, across all the samples in the dataset. Phylogenetic diversity (Faith’s PD) (Faith, 1992) was estimated using the function estimate_pd in package “btools” (Battaglia, 2021). “Phyloseq” was also used for non-metric multidimensional scaling (NMDS) using Bray–Curtis dissimilarity matrices to visualize community patterns. The “vegan” (Oksanen et al., 2018) function envfit was used to correlate chemical and taxonomical data with the community structure and the function adonis to test differences in the community structures by permutational multivariate analysis of variance (PERMANOVA, using nperm = 999). Pairwise testing after significant PERMANOVAs was done using function pairwise.perm.manova from the package “RVAideMemoire” (Hervé, 2020; 999 permutations and *p*-value adjustment with “false discovery rate”). The alpha diversity estimates were done using non-rarefied data, and all other analyses were done using rarefied data. Student’s *t*-test (assuming unequal variances and two-sided, if not indicated else) was used when comparing two groups, and Tukey’s honest significance test (HSD test) was used when comparing three or more groups (α = 0.05). Gene abundance data were log-transformed before statistical testing. Statistical analyses were carried out in R, version 4.0.3 (R Development Core Team, 2016).

## Results

### Water Chemistry and N Removal

**FIGURE 2:**
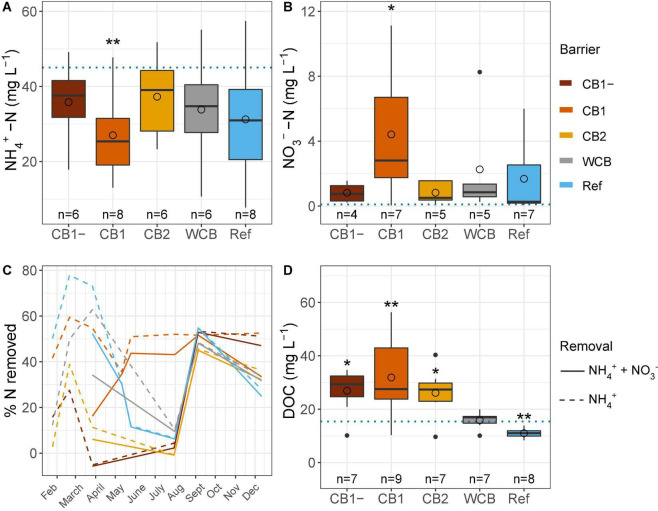
Water chemistry after passage through the reactive barriers and reference across all time points. **(A)** Ammonium concentration, **(B)** nitrate concentration, **(C)** removal of total reactive nitrogen and ammonium over time, and **(D)** concentration of dissolved organic carbon. Inflow concentrations are indicated with dotted lines in panels **(A,B,D)** (mean values over the year, *n* = 31, 29, and 12 for ammonium, nitrate, and dissolved organic carbon, respectively). Box limits represent the inter-quartile range with median values represented by the center line and mean values by circles. Whiskers represent values ≤ 1.5 times the upper and lower quartiles, while points indicate values outside this range. The number of samples per barrier is indicated below the boxes. Stars denote if the mean value is significantly different from the mean inflow value (*t*-test, *p < 0.05, **p < 0.01).

Regardless of the barrier composition, ammonium concentrations were generally reduced and nitrate concentrations were increased after passage through the barrier, albeit significantly only in the CB1 with vegetation in the barrier (Figures 2A,B). CB1 showed the highest removal of ammonium from mid-May and throughout the experimental period, and despite the production of nitrate, the removal of reactive N was more than 40% during summer (Figure 2C). From 23 May to 29 August, the concentration of dissolved oxygen was lower than observed during the rest of the year (15.1 ± 5.3 and 50.1 ± 19.7%, respectively, mean ± SD, *t*-test, *p* < 0.001), which coincided with lower ammonium removal. The high and similar N removal capacity in all the barriers at the beginning of September might be caused by the of high dissolved oxygen concentration in the inflow water at that time (Supplementary Table 1). When normalizing ammonium removal to the removal capacity of the reference, CB1 had a 2.3 times higher capacity, but CB1-, CB2, and WCB removed less, 0.39, 0.39 and 0.70 times the reference, respectively (mean values of ratios of % reactive N removal, *n* = 4–8). Over the year, the amount of reactive N removed was moderate, with an average of 29% across all the barriers (including the reference, which showed a 29% removal), and there were no differences between the barriers (HSD test, Supplementary Figure 2). The DOC concentration increased in the water after passing through the compost barriers, but was found to be reduced in the reference and unaffected in the WCB groups (Figure 2D).

### Genetic Potential for N-Transformation Processes

**FIGURE 3:**
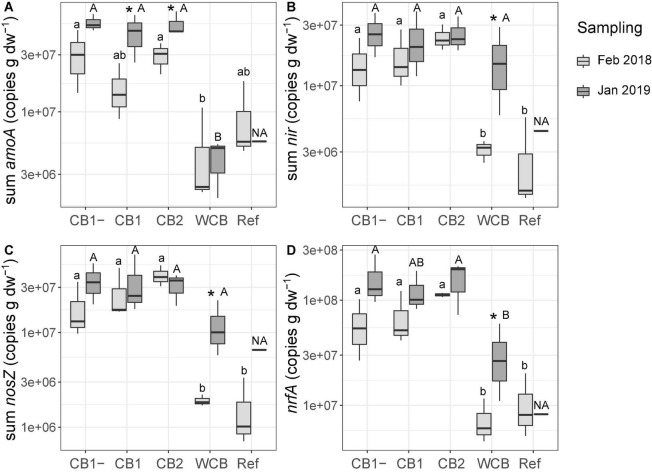
Genetic potential for **(A)** ammonia oxidation, **(B)** denitrification, **(C)** nitrous oxide reduction, and **(D)** DNRA in the barrier materials and reference with top sand excluded across all depths based on the sum of functional groups for each process. Box limits represent the inter-quartile range with median values represented by the center line and whiskers represent values ≤ 1.5 times the upper and lower quartiles. For each barrier, stars denote if the mean values of the final abundances are significantly higher than the initial abundances (one-sided *t*-test, *p < 0.05, *n* = 3, reference final sampling *n* = 1). Different small letters above boxes indicate significant differences between barriers at the initial sampling and capital letters indicate differences at the final sampling occasion (Tukey’s HSD test, α = 0.05, and the final sampling in the reference is not included in the test, denoted NA).

Gene abundances in the barrier material are shown separately for the top sand and the actual barrier layers in Supplementary Figure 3. The abundances in the top sand were lower than those in the reactive layer of all the compost barriers. With few exceptions, the gene abundances in the compost and woodchip barriers were higher after the fourth wet cycle compared to those observed after the first wet cycle, whereas the reference displayed increases only for *nirS* and *nosZ*I. The potential for ammonia oxidation (the sum of archaeal and bacterial *amoA* gene abundances), increased in the two compost barriers having vegetation (Figure 3A; one-sided *t-*test, top sand excluded, *p* < 0.05). In the woodchip barrier, there was an increase in the genetic potential for denitrification (sum of *nir* genes), nitrous oxide reduction (sum of *nosZ* genes), and DNRA (*nrfA*) (Figures 2B–D; one-sided *t-*test, top sand excluded, *p* < 0.05). Possible changes in the reference could not be tested statistically due to missing samples at the final sampling stage. A week into the first dry cycle, all processes but nitrification had a higher genetic potential in the compost barriers than in the WCB and reference (Figures 3A–D; HSD test). In the end, a week into the fourth dry cycle, denitrification and nitrous oxide reduction potentials were similar among all the treatments, while nitrification potential remained higher in the CB barriers than in the WCB barrier, reference excluded (Figures 3A–C; HSD test). The ratio between DNRA and nitrification [*nrfA/*(bacterial *amoA* + archaeal *amoA*)] was significantly lower at the end of the experiment in the vegetated CB1 barrier (not shown, *t*-test *p* = 0.025).

The genetic potentials of the N-transforming processes in the water were determined during the fourth wet cycle. Gene abundances involved in nitrous oxide reduction, DNRA, and denitrification (*nirK*) were higher in the water collected from the woodchip barrier when compared to water collected from the reference and compost barriers. The other gene abundances in the water showed no specific pattern (Supplementary Table 1).

### Diversity and Community Structure

**FIGURE 4:**
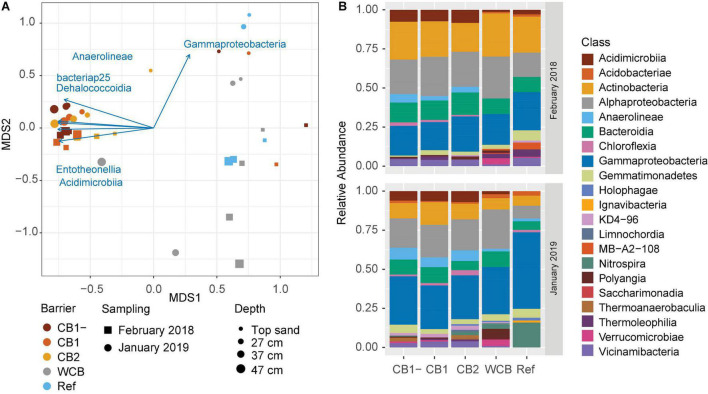
Structure and taxonomic composition of microbial communities in the reactive barrier material. **(A)** Ordination is based on non-metric multidimensional scaling of Bray–Curtis dissimilarities of OTU abundances (stress = 0.13). Taxonomic classes that significantly correlated with the ordination (*p* < 0.05 and r^2^ > 0.7) are shown as vectors. The lengths of the vectors are proportional to the strength of the correlation. **(B)** Mean relative abundances across the depths of microbial classes (>2% of the community) in the barriers at the initial and final sampling stages.

The phylogenetic diversity (Faith’s PD) initially differed between CB2 and WCB samples, but after 1 year, there were no significant differences among the barriers (Supplementary Figure 4A; HSD test). Shannon’s entropy and Pielou’s evenness showed the same pattern (Supplementary Figures 4B,C; HSD test). Differences in the structure of microbial community between samples were mainly driven by barrier composition and sampling occasion (PERMANOVA, *p* = 0.001 and 0.003, respectively; Figure 4A). Two groups could be distinguished: one group included the compost barriers and the other included WCB and reference (pairwise comparisons after PERMANOVA, *p* > 0.16 within groups and *p* < 0.007 between the two groups). The top sand in the compost barriers was, however, more similar to the WCB and reference samples than to the deeper layers of compost barriers (Figure 4A). Actinobacteria, Alphaproteobacteria, and Gammaproteobacteria dominated the barrier samples both at the beginning and the end of the experimental period (Figure 4B). In total, 77 classes (36 phyla) were represented in the barriers, and most of them were significantly correlated with the community structure (Supplementary Table 3). Higher abundances of Gammaproteobacteria were correlated with the end point samples (Figures 4A,B), but most of the bacterial classes correlating to the separation of community structure were strongly associated (r^2^ > 0.7) with the compost barriers (Figure 4A and Supplementary Table 3). Nitrospira was the second most abundant class present in the reference at the final sampling stage (Figure 4B). Organic carbon content and pH also correlated with the separation of the communities; organic carbon correlated with the compost barriers and samples from start, and pH with the deeper layers in the WCB (Supplementary Table 3; *p* = 0.019 and 0.011, respectively).

All gene abundances correlated positively with the compost barrier communities. Differences in the correlations with start and end communities were seen for *nir* genes, where *nirK* and *nirS* correlated with those from start and end, respectively. Also, *nosZ*I correlated with end samples, whereas *nosZ*II showed no pattern with sampling occasion (Supplementary Table 3).

Although the water samples were also dominated by Actinobacteria and Gammaproteobacteria (Supplementary Figure 5), the water and barrier samples had distinctly different bacterial communities (PERMANOVA, *p* = 0.001) at the end of the experimental period. Other highly represented classes in the water samples were Bacteroidia, Campylobacteria, and Clostridia. The water from the WCB also had a high occurrence of Desulfobulbia and Desulfuromonadia, classes not found in the water sampled from the other barriers or in the barrier samples (Supplementary Figure 5). The water from the WCB had the highest diversity and evenness, as indicated by Shannon’s and Pielou’s indices (Supplementary Figures 4B,C). Overall, the water communities had a lower diversity based on Shannon’s entropy than the barrier communities across time and among barrier types (Supplementary Figure 4B), but the phylogenetic diversity did not differ between water and barrier samples (Supplementary Figure 4A).

## Discussion

The overall N removal performance of the barriers was moderate, with only 29% of N removed on average across all the barrier types. During the period with less dissolved oxygen concentrations available in the water fed to the barriers, removal of ammonium decreased in all barriers except for CB1, which can likely be attributed to oxygen deficiency for the organisms involved in nitrification. We can only speculate why CB1 showed a more stable performance and functioned better than the other barriers under oxygen-limiting conditions. Compared to CB1-, CB1 had vegetation. The idea behind having vegetation in the barriers was to prevent clogging by facilitating the flow through small channels made by the roots. In addition, small channels might have helped in the advection and diffusion of oxygen, thereby favoring nitrification. However, this phenomenon should also have occurred in CB2. The higher proportion of compost, likely increasing heterotrophic respiration and thereby decreasing oxygen levels, is a more plausible explanation for the differences in ammonium removal. The significant removal of ammonium in the CB1 barrier with vegetation did not lead to a significantly higher net removal of reactive N in the barrier, since nitrate was formed. Nevertheless, decreasing the ammonium load is still advantageous, not only because of its toxicity, but also because it contributes to decreasing biological oxygen demand, thus preventing anoxic conditions in the recipient aquifer. Allowing plants to grow in the barriers could have several effects on the N dynamics, the most obvious being plant N uptake and accumulation in plant biomass. This could contribute to the lowered ammonium concentrations in the water from CB1 in comparison to the corresponding barrier without plants (CB1-). However, plants can also affect microbial activity, for example, resulting in immobilization of available ammonium (Skiba et al., 2011), and either increase or decrease nitrification depending on the plant species and soil (Skiba et al., 2011; Thion et al., 2016; Norton and Ouyang, 2019). Although our experimental design does not allow us to decipher the mechanisms, allowing plants to grow in the barriers makes the system resemble a vertical sub-surface flow constructed wetland or reedbed (Vymazal, 2007; Al-Ajalin et al., 2020), a design that has proven to be efficient in the removal of nutrients and organic material from domestic wastewater (e.g., Blankenberg et al., 2008). Further, in a full-scale SAT with more frequent alteration of wet and dry periods (Sharma and Kennedy, 2017), additional nitrification in the vadose zone, together with dilution when the recharged water reaches the aquifer, will contribute to lower ammonium levels in the aquifer than measured in our pilot system (Sopilniak et al., 2017).

The composition of the bacterial community differed and largely reflected the concentrations of DOC in the water after passage through the barrier, but in contrast to our hypothesis about diversity, there were no differences between the reactive barriers at the end of the first year of operation (reference barrier excluded). The reference showed a relative increase in Nitrospira, which is known to be involved in nitrification and is important in sand filters (Fowler et al., 2018), yet this was not reflected in the ammonium removal. The presence of compost in the barriers was the strongest driver of community structure, and hence strengthens the part of our first hypothesis about different communities developing in the barriers and supports the lab-scale study using the same compost material (Modrzyński et al., 2021). The type of available carbon is known to be an important factor that determines the development of soil bacterial communities (e.g., Cederlund et al., 2014), as well as their metabolic capacity and N removal rates in wastewater treatment plants (e.g., Hallin et al., 2006). One of the bacterial classes present in higher abundance in the compost barriers, which also increased in relative abundance over the year, was Anaerolineae, belonging to the phylum Chloroflexi. It has been retrieved from a wide range of habitats (Yamada and Sekiguchi, 2009), and members of this class have genes for cellulose hydrolyzation (Xia et al., 2016). The abundance of uncharacterized Chloroflexi KD4-96 also increased in the barriers with time and has previously been found in polluted soils (Gołębiewski et al., 2014; Wegner and Liesack, 2017). The origin of the compost used in our experiment was based on waste from gardens, and compost of a different origin could potentially promote a different community with other carbon degradation pathways or tolerance and capacity to degrade contaminants. Thus, when designing multipurpose reactive barriers, the origin of the compost could be a way of controlling which microorganisms proliferate and thrive in the barriers.

The differences in the genetic potentials for N-transforming processes that developed during the first year of operation were not reflected in the N removal activity among the barriers. The poor ammonium removal in the compost barriers could be due to the observed trend of increasing genetic potential for DNRA, even though we detected a high abundance and significant increase in ammonia-oxidizing bacteria and archaea in these barriers. A previous study has reported an increase in the DNRA bacteria in the lower, anoxic layers in compost-based barriers, resulting in increased ammonium production despite efficient nitrification in the upper, oxic barrier layers (Modrzyński et al., 2021). The exception in our study was the increased ammonium removal in CB1, which had a lower ratio between *nrfA* (DNRA) and *amoA* (ammonia oxidation) in the barrier material at the end of the experimental period. Another aspect when comparing *amoA* abundances between the compost barriers *versus* the woodchips and reference is the possible effect of N mineralization. We speculate that N mineralization of organic matter in the compost continuously supplies the systems with ammonium (Habteselassie et al., 2006), and thereby supports the development of a larger ammonia-oxidizing community in the compost barriers. Hence, the resulting ammonium levels in the water after barrier passage is not lower in the compost barriers despite significantly higher *amoA* abundances. In other woodchip-based systems for N removal, it has been shown that changes in the microbial community composition appear within a few months (Grießmeier et al., 2017; Aalto et al., 2020; Hellman et al., 2021; Jéglot et al., 2021). Given that the increase in copy numbers of *nir* and *nosZ* genes in the woodchip barrier resulted in the same genetic potential for denitrification and nitrous oxide reduction between the compost and woodchip barriers, it is plausible that in the present systems, the potentials for denitrification and nitrous oxide reduction were similar among the barriers during a substantial part of the experimental period, which contributed to the equal overall removal of reactive N. A higher temporal resolution would be needed to better understand this phenomenon.

To conclude, our study found small or no differences in ammonium or overall N removal among the barriers, despite large differences in the composition of microbial community and genetic potential for N-transforming processes. The barrier with vegetation and the highest proportion of compost continued to remove approximately 50% of the ammonium even under conditions with low oxygen concentration in the water. The composition of the barrier material will be crucial to achieve sufficient N removal and avoid leakage of DOC. In this regard, a barrier based on woodchips is preferred over compost, but the removal of other pollutants must be considered as well, indicating that designing reactive barriers for multiple purposes is a challenge. In summary, a range of materials is likely necessary to achieve sustainable urban wastewater management using a soil aquifer treatment system in combination with barriers when recharging aquifers.

## Data Availability Statement

The raw sequence dataset is available under the BioProject accession number PRJNA773712 in the NCBI sequence read archive (SRA) at https://www.ncbi.nlm.nih.gov/. The other data used in the analyses are in the Supplementary Table 1.

## Author Contributions

LM-L, CV, JC, and SH designed the study. LM-L and CV conducted the fieldwork. MH performed the laboratory work. MH and JJ analyzed the data. MH, SH, and JJ prepared the manuscript, with input from the other authors. All authors approved the final version of the manuscript for publication.

## Conflict of Interest

The authors declare that the research was conducted in the absence of any commercial or financial relationships that could be construed as a potential conflict of interest.

## Publisher’s Note

All claims expressed in this article are solely those of the authors and do not necessarily represent those of their affiliated organizations, or those of the publisher, the editors and the reviewers. Any product that may be evaluated in this article, or claim that may be made by its manufacturer, is not guaranteed or endorsed by the publisher.
