# Supplementary material for: Nitrogen Removal Capacity of Microbial Communities Developing in Compost- and Woodchip-Based Multipurpose Reactive Barriers for Aquifer Recharge With Wastewater
Source: Front Microbiol. 2022 May 24;13:877990. doi: 10.3389/fmicb.2022.877990 (PMC9171435; doi:10.3389/fmicb.2022.877990)
Supplement: Supplementary file 1 [file Data_Sheet_1.PDF]

Supplementary material for

Nitrogen removal capacity in microbial communities developing in compost- and woodchip-based multipurpose reactive barriers for aquifer recharge with wastewater

Maria Hellman[1]*, Cristina Valhondo[2,3], Lurdes Martínez-Landa[3,4], Jesús Carrera[2,3], Jaanis Juhanson[1], Sara Hallin[1]

[1]Department of Forest Mycology and Plant Pathology, SLU Uppsala, Sweden

[2]Institute of Environmental Assessment and Water Research (IDAEA), CSIC, Barcelona, Spain

[3]Hydrogeology Group (UPC-CSIC), Associate Unit, Barcelona, Spain

[4]Department of Civil and Environmental Engineering, Universitat Politecnica de Catalunya (UPC), Barcelona, Spain

Figures

Figure S1. Influent water concentrations of nitrate, ammonium and dissolved organic carbon 2018.

Figure S2. Removal of reactive N.

Figure S3. Genetic potential for nitrogen transformation processes in top sand and barrier material

Figure S4. Alpha diversity across all barrier and water samples.

Figure S5. Relative contribution of microbial classes in the water samples.

Tables

Table S1. Location, sampling date, chemical and microbial characterization of all samples.

Table S2. Primers and reaction conditions for qPCR.

Table S3. Results from vegan function envfit.

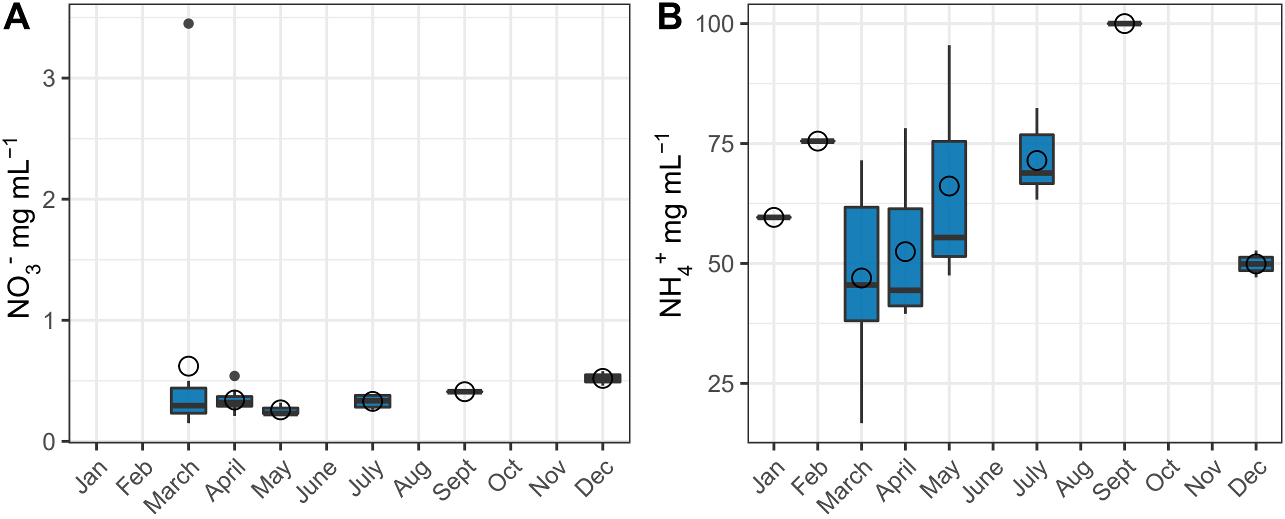

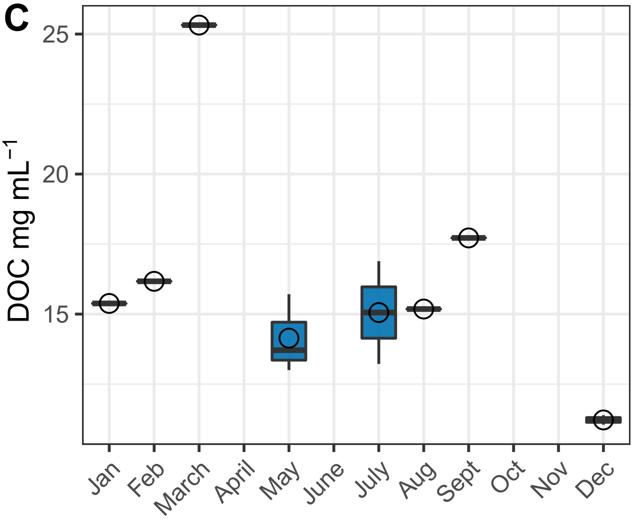

Figure S1. Concentrations of (**A**) nitrate (n = 1 – 10), (**B**) ammonium (n = 1 – 10) and (**C**) dissolved organic carbon (n = 1 – 3) in the influent water 2018. Box limits represent the inter-quartile range with median values represented by the center line and mean values by circles. Whiskers represent values ≤ 1.5 times the upper and lower quartiles, while points indicate values outside this range.

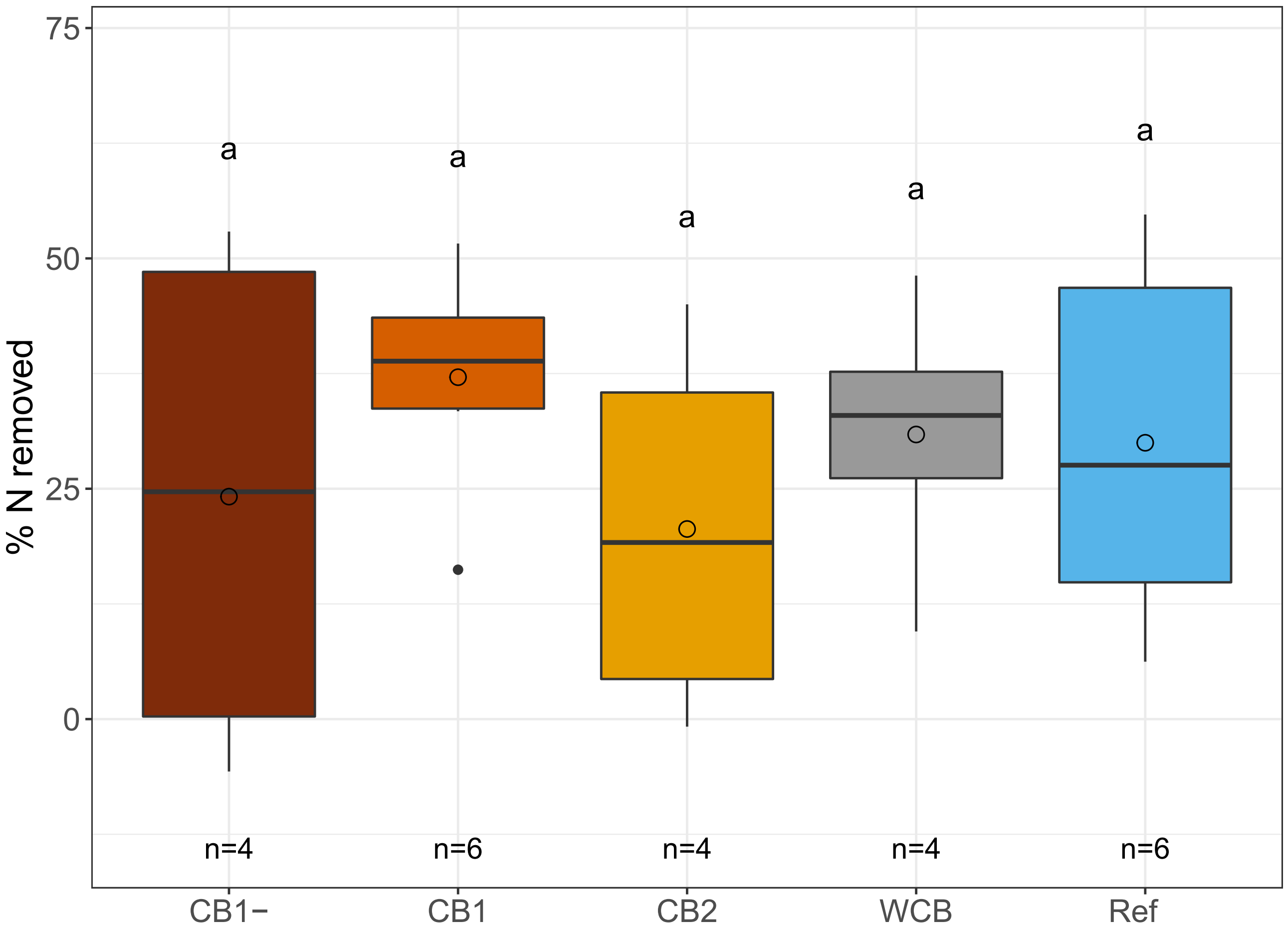

Figure S2. Reactive nitrogen removed in the water after passage through the reactive barriers and reference across all time points. Box limits represent the inter-quartile range with median values represented by the center line and mean values by circles. Whiskers represent values ≤ 1.5 times the upper and lower quartiles, while points indicate valuesoutside this range. Number of samples per barrier is indicated below the boxes.

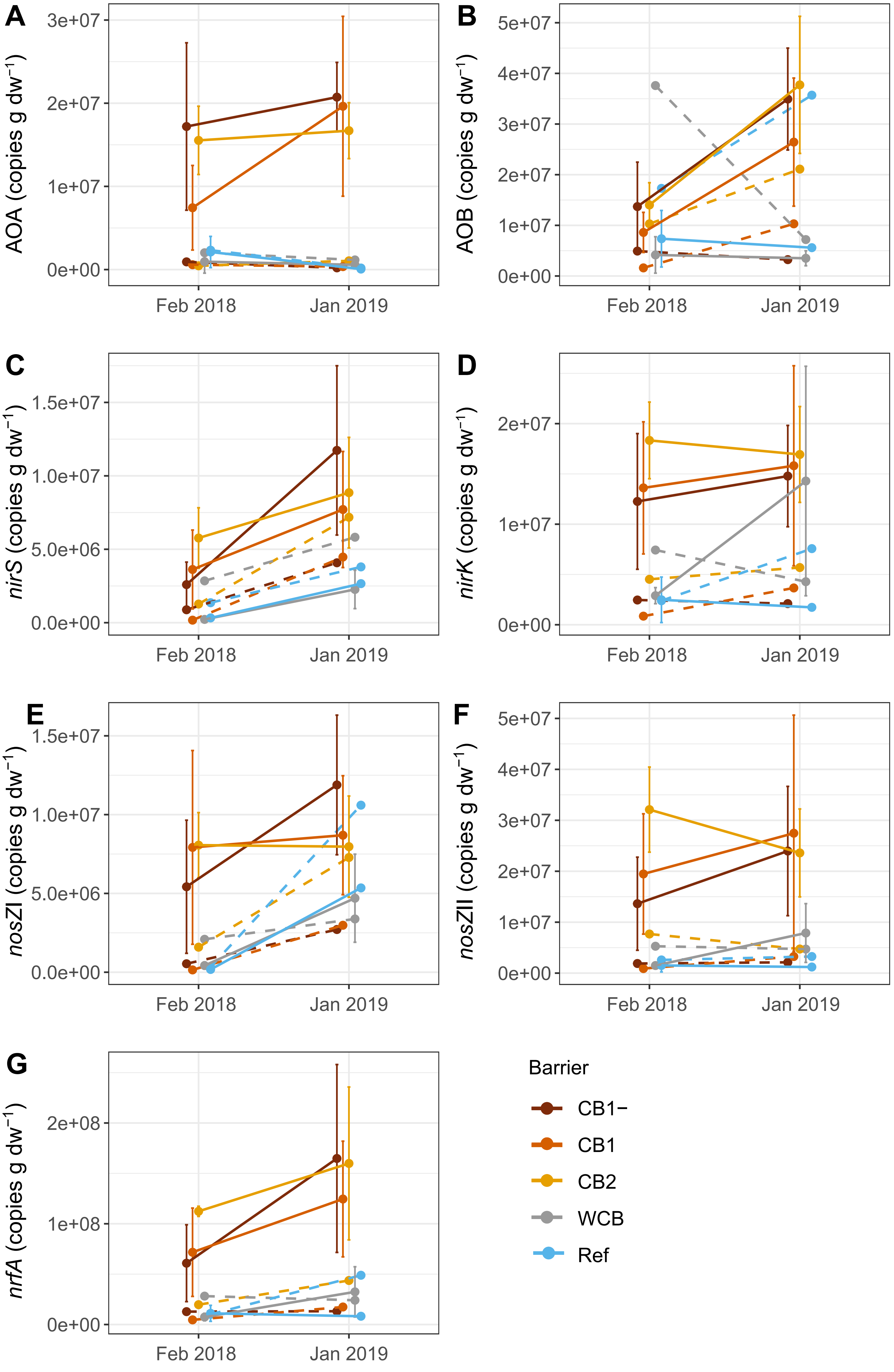

Fig. S3. Genetic potential for nitrogen transformation processes in top sand and barrier material shown as individual gene abundances at the initial (Feb 2018) and final (Jan 2019) sampling point. (**A**) *amoA* in ammonia oxidizing archaea, (**B**) *amoA* in ammonia oxidizing bacteria, (**C**) *nirS* in denitrifiers, (**D**) *nirK* in denitrifiers, (**E)** *nosZ*I in denitrifiers/nitrous oxide reducers, (**F**) *nosZ*II in denitrifiers/nitrous oxide reducers, (**G**) *nrfA* in DNRA bacteria. Dashed lines indicate gene abundances in the top sand and solid lines connect points representing the mean value of abundances at three depths (27, 37 and 47 cm) at each sampling occasion. Error bars show SD, n = 3 (Ref final sampling, n=1).

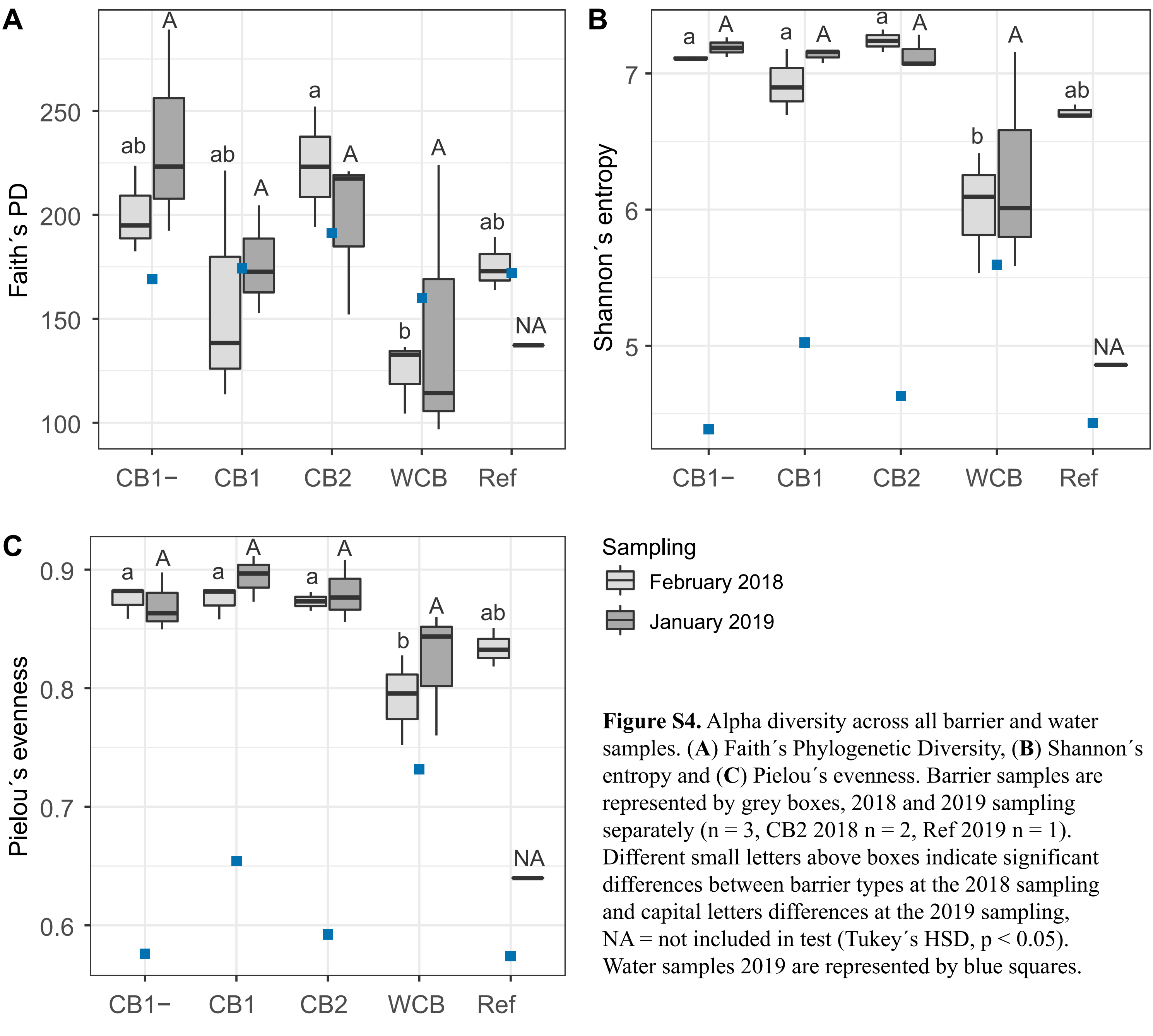

Figure S4. Alpha diversity across all barrier and water samples. (**A**) Faith´s Phylogenetic Diversity, (**B**) Shannon´s entropy and (**C**) Pielou´s evenness. Barrier samples are represented by grey boxes, 2018 and 2019 sampling separately (n = 3, CB2 2018 n = 2, Ref 2019 n = 1). Different small letters above boxes indicate significant differences between barrier types at the 2018 sampling and capital letters differences at the 2019 sampling, NA = not included in test (Tukey´s HSD, $p < 0.05$). Water samples 2019 are represented by blue squares.

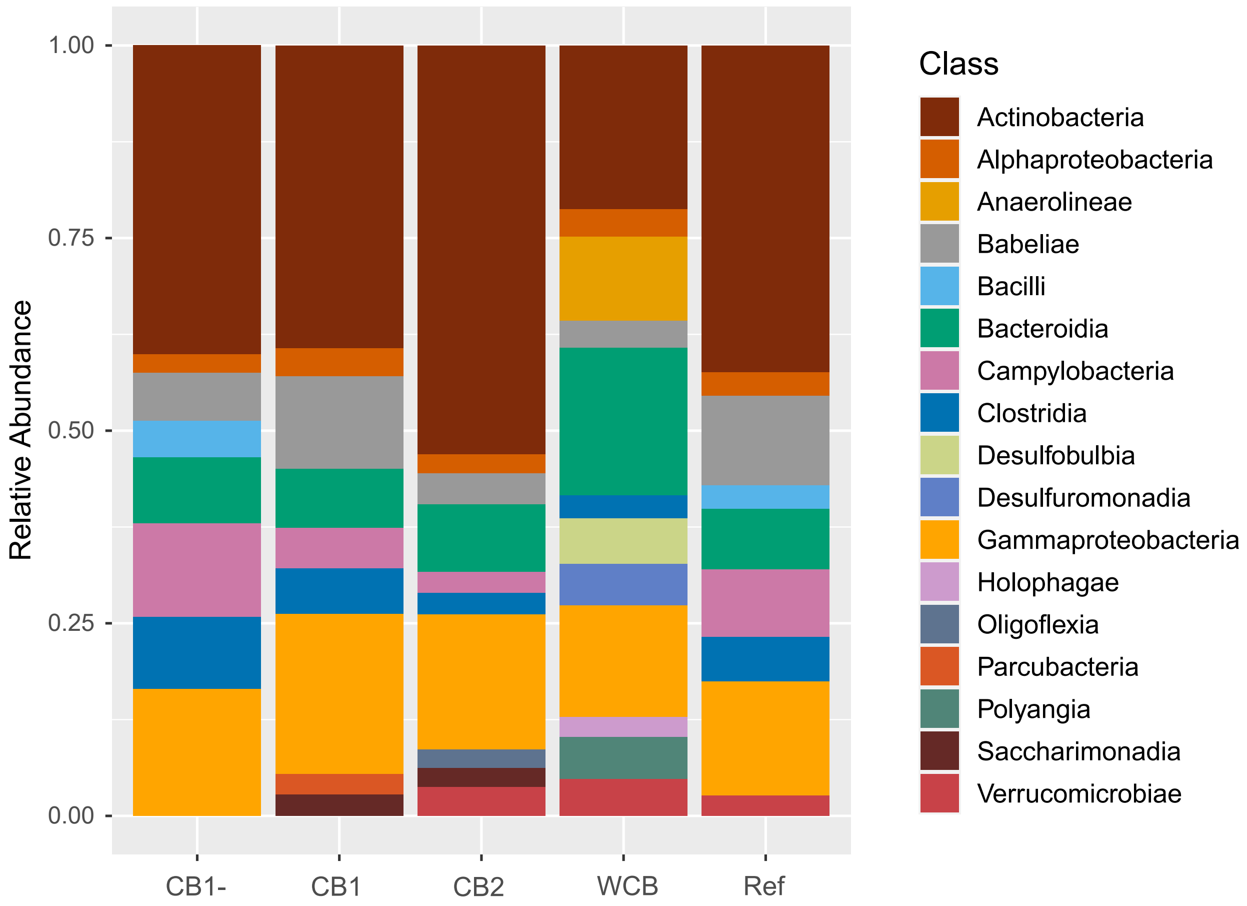

Figure S5. Relative contribution of microbial classes (> 2 % of the community) in the water samples (Dec 2018).

Table S1. Location, sampling date, chemical and microbial characterization of all samples.

Sample type	Barrier or Inflow	Date	Sampling	Depth cm	pH	LOI % of dw	NH4 mg L-1	NO3 mg L-1	DOC mg L-1	Oxygen mg L-1	Oxygen %	Faith PD	Shannon	Pielou	16S r RNA g cop g dw-1 cop L-1	AOA cop g dw-1 cop L-1	AOB cop g dw-1 cop L-1	*nirS* cop g dw-1 cop L-1	*nirK* cop g dw-1 cop L-1	*nosZ* I cop g dw-1 cop L-1	*nosZ* II cop g dw-1 cop L-1	*nrfA* cop g dw-1 cop L-1
Water	INF	2018-01-25			7.9		59.6		15.38	6.2	61											
Water	INF	2018-02-20			7.9		75.5		16.17													
Water	INF	2018-03-06			7.9		46.7	0.5	25.32													
Water	INF	2018-03-07			7.4		40.9	0.44		3.7												
Water	INF	2018-03-23					71.5	0.33														
Water	INF	2018-03-25			7.1		16.7	3.45		4.4	40											
Water	INF	2018-03-26			7.3		27.1	0.44		4.5	43											
Water	INF	2018-03-27			7.3		37.1	0.23		2.4	23											
Water	INF	2018-03-28			7.3		44.3	0.26		1.2	13											
Water	INF	2018-03-29			7.3		59.4	0.24		1.0	10											
Water	INF	2018-03-30			7.5		63.4	0.15		5.5	56											
Water	INF	2018-03-31			7.5		62.5	0.17		5.6	58											
Water	INF	2018-04-01			7.6		44.4	0.42		5.4	54											
Water	INF	2018-04-02			7.9		42.0	0.3		7.8	80											
Water	INF	2018-04-03			7.7		46.3	0.28		6.0	64											
Water	INF	2018-04-04			7.6		40.3	0.54		5.3	55											
Water	INF	2018-04-05			7.6		39.5	0.21		6.3	64											
Water	INF	2018-04-06			7.7		78.2	0.32		6.4	68											
Water	INF	2018-04-07			7.6		76.5	0.32		5.9	60											
Water	INF	2018-05-11			7.7		95.5	0.23	13.00	5.9	69											
Water	INF	2018-05-23			7.7		55.4	0.23	13.71	0.8	9.3											
Water	INF	2018-05-25			7.7		47.5	0.32	15.71	0.6	7.1											
Water	INF	2018-07-20			7.6		63.3	0.32		1.3	16											
Water	INF	2018-07-24			7.6		82.4	0.26		1.5	18											
Water	INF	2018-07-25					69.7	0.39														
Water	INF	2018-07-27			7.7		68.0	0.39		1.4	18											
Water	INF	2018-07-30			7.7		66.2	0.35	13.22	1.0	13											
Water	INF	2018-07-31			7.6		79.2	0.27	16.89	1.2	15											
Water	INF	2018-08-29			7.7				15.18	1.9	24											
Water	INF	2018-09-05			7.2		100.0	0.41	17.72	5.2	65											
Water	INF	2018-12-10			7.3		47.1	0.58	11.05	3.0	30											
Water	INF	2018-12-11			7.3		52.7	0.46	11.39	3.8	38											
Water	CB1-	2018-01-25			7.7		50.2		34.69	0.2	1.6											
Water	CB1-	2018-02-01							29.34													
Water	CB1-	2018-02-01							28.46													
Water	CB1-	2018-02-20			7.4		54.7		31.42	1.6	15											
Water	CB1-	2018-03-27			7.2		39.0	0.97		0.1	0.8											
Water	CB1-	2018-07-30			7.2		63.3	5.13	33.43	0.8	10											
Water	CB1-	2018-09-05			7.2		46.7	1.52	20.89	0.8	10											
Water	CB1-	2018-12-10	final		6.8		23.0	6.92	10.15	1.9	18	169	4.386	0.576	3.78E+08	7.88E+06	4.08E+06	3.21E+06	2.77E+06	2.28E+06	4.34E+06	2.47E+07
Water	CB1	2018-01-25			7.3		34.8	12.43	56.34	0.2	1.9											
Water	CB1	2018-02-01							42.96													
Water	CB1	2018-02-01							43.16													
Water	CB1	2018-02-20			7.2		30.6		37.31	0.3	2.8											
Water	CB1	2018-03-27			6.7		16.8	49.25		0.4	3.8											
Water	CB1	2018-05-11			6.8		61.5	3.53	23.78	0.6	6.1											
Water	CB1	2018-05-25			6.8		23.3	11.97	25.85	0.3	3.7											
Water	CB1	2018-07-31			6.8		38.0	24.37	27.52	0.6	7.5											
Water	CB1	2018-09-05			7.4		48.4	0.18	19.65	0.4	5.3											
Water	CB1	2018-12-11	final		6.6		25.0	34.97	10.3	0.8	7.8	174	5.023	0.654	3.92E+08	3.84E+06	1.91E+06	4.00E+06	2.72E+06	1.77E+06	5.80E+06	2.39E+07
Water	CB2	2018-01-25			7.6		57.9	1.63	27.38	0.2	1.9											
Water	CB2	2018-02-01							28.22													
Water	CB2	2018-02-01							31.37													
Water	CB2	2018-02-20			7.0		46.2		40.34	0.6	5.8											
Water	CB2	2018-03-27			6.8		32.9	6.9		0.2	1.3											
Water	CB2	2018-07-30			7.3		66.7	0.51	22.63	0.6	7.0											
Water	CB2	2018-09-05			7.1		54.4	2.24	23.31	0.3	4.0											
Water	CB2	2018-12-10	final		7.0		30.0	6.94	9.65	0.9	9.1	191	4.631	0.592	3.21E+08	5.60E+06	6.38E+05	2.15E+06	3.50E+06	1.73E+06	6.32E+06	3.14E+07
Water	WCB	2018-01-25			7.6		52.3	5.99	16.96	0.2	1.9											
Water	WCB	2018-02-01							15.49													
Water	WCB	2018-02-01							19.94													
Water	WCB	2018-02-20			7.0		37.9		17.08	0.6	5.8											
Water	WCB	2018-03-27			6.8		13.8	36.56		0.2	1.3											
Water	WCB	2018-07-31			7.0		71.0	2.54	14.04	0.4	5.6											
Water	WCB	2018-09-05			7.1		51.6	1.14	17.5	0.3	4.0											
Water	WCB	2018-12-11	final		6.7		35.0	3.75	10.09	0.6	6.5	160	5.595	0.731	7.74E+08	4.22E+05	1.49E+06	4.93E+06	7.25E+06	3.31E+06	2.41E+07	8.93E+07
Water	Ref	2018-01-25			7.1		29.7	16.58	10.71	0.2	2.1											
Water	Ref	2018-02-01							9.21													
Water	Ref	2018-02-20			7.1		16.7		10.16	0.9	8.2											
Water	Ref	2018-03-27			6.5		9.99	26.54		0.4	4.0											
Water	Ref	2018-05-11			7.0		66.5	0.49	11.33	0.3	3.6											
Water	Ref	2018-05-25			7.0		41.8	1.09	11.66	0.3	3.6											
Water	Ref	2018-07-31			7.1		74.0	1.17	13.87	0.5	6.6											
Water	Ref	2018-09-05			7.3		45.2	0.32	12.69	0.7	8.3											
Water	Ref	2018-12-11	final		6.7		38.0	5.87	8.37	0.9	8.8	172	4.433	0.574	2.65E+08	2.14E+06	1.39E+06	1.77E+06	3.10E+06	2.07E+06	5.46E+06	3.31E+07
Barrier	CB1-	2018-02-08--09	initial	Top sand	7.8	0.39						118	5.384	0.72	2.24E+08	9.27E+05	4.92E+06	8.81E+05	2.46E+06	5.38E+05	1.89E+06	1.28E+07
Barrier	CB1-	2018-02-08--09	initial	27	7.6	4.84						195	7.125	0.883	4.04E+08	1.20E+07	1.80E+07	1.90E+06	1.14E+07	3.01E+06	1.01E+07	5.39E+07
Barrier	CB1-	2018-02-08--09	initial	37	7.6	5.70						182	7.102	0.882	1.12E+09	2.88E+07	1.95E+07	4.35E+06	1.94E+07	1.03E+07	2.40E+07	1.02E+08
Barrier	CB1-	2018-02-08--09	initial	47	7.9	4.71						224	7.11	0.858	2.82E+08	1.08E+07	3.61E+06	1.53E+06	5.99E+06	2.96E+06	6.78E+06	2.67E+07
Barrier	CB1	2018-02-08--09	initial	Top sand	7.7	0.34						118	6.079	0.791	4.70E+07	5.83E+05	1.60E+06	1.73E+05	8.55E+05	1.47E+05	8.98E+05	4.63E+06
Barrier	CB1	2018-02-08--09	initial	27	7.9	3.36						138	6.898	0.881	1.24E+09	1.33E+07	1.22E+07	6.67E+06	2.10E+07	1.50E+07	3.31E+07	1.22E+08
Barrier	CB1	2018-02-08--09	initial	37	7.5	4.45						114	6.693	0.884	4.74E+08	4.64E+06	9.24E+06	2.60E+06	1.14E+07	4.81E+06	1.26E+07	5.17E+07
Barrier	CB1	2018-02-08--09	initial	47	7.7	5.18						221	7.181	0.858	4.08E+08	4.36E+06	4.36E+06	1.60E+06	8.43E+06	3.95E+06	1.27E+07	4.15E+07
Barrier	CB2	2018-02-08--09	initial	Top sand	7.3	0.58						189	6.539	0.807	4.88E+08	4.53E+05	1.03E+07	1.27E+06	4.52E+06	1.59E+06	7.68E+06	1.97E+07
Barrier	CB2	2018-02-08--09	initial	27	7.5	3.62						252	7.322	0.865	9.81E+08	1.54E+07	1.52E+07	6.08E+06	1.66E+07	8.49E+06	3.00E+07	1.07E+08
Barrier	CB2	2018-02-08--09	initial	37	7.6	2.68						194	7.157	0.881	1.26E+09	1.97E+07	1.77E+07	7.65E+06	2.27E+07	9.88E+06	4.13E+07	1.17E+08
Barrier	CB2	2018-02-08--09	initial	47	7.6	4.80									8.49E+08	1.15E+07	9.15E+06	3.57E+06	1.57E+07	5.82E+06	2.50E+07	1.13E+08
Barrier	WCB	2018-02-08--09	initial	Top sand	7.3	0.62						143	5.976	0.763	3.87E+08	2.02E+06	3.76E+07	2.85E+06	7.43E+06	2.09E+06	5.28E+06	2.82E+07
Barrier	WCB	2018-02-08--09	initial	27	7.8	0.49						136	6.414	0.828	8.21E+07	2.54E+06	8.30E+06	4.70E+05	2.00E+06	1.95E+05	1.64E+06	1.16E+07
Barrier	WCB	2018-02-08--09	initial	37	7.8	7.80						133	6.094	0.795	6.18E+07	2.30E+05	1.90E+06	9.97E+04	3.14E+06	4.31E+05	1.27E+06	6.04E+06
Barrier	WCB	2018-02-08--09	initial	47	7.7	12.1						104	5.533	0.752	1.09E+08	7.96E+04	2.27E+06	1.02E+05	3.54E+06	6.49E+05	1.56E+06	4.55E+06
Barrier	Ref	2018-02-08--09	initial	Top sand	7.5	0.40						136	6.04	0.777	1.01E+08	2.33E+06	1.73E+07	1.36E+06	2.39E+06	3.38E+05	2.59E+06	1.01E+07
Barrier	Ref	2018-02-08--09	initial	27	7.8	0.40						173	6.69	0.832	1.22E+08	4.26E+06	1.38E+07	5.40E+05	5.09E+06	3.56E+05	2.98E+06	2.01E+07
Barrier	Ref	2018-02-08--09	initial	37	8.1	0.35						189	6.678	0.818	2.88E+07	8.15E+05	3.91E+06	1.60E+05	1.14E+06	6.86E+04	6.42E+05	5.10E+06
Barrier	Ref	2018-02-08--09	initial	47	8.1	0.38						164	6.772	0.851	4.13E+07	1.26E+06	4.36E+06	2.76E+05	1.21E+06	1.02E+05	9.15E+05	8.11E+06
Barrier	CB1-	2019-01-14	final	Top sand	7.4	0.50						170	6.269	0.794	8.88E+07	2.07E+05	3.23E+06	4.08E+06	2.10E+06	2.71E+06	2.12E+06	1.32E+07
Barrier	CB1-	2019-01-14	final	27	7.3	5.24						223	7.121	0.863	6.10E+08	1.61E+07	3.70E+07	7.51E+06	9.25E+06	7.35E+06	1.25E+07	9.64E+07
Barrier	CB1-	2019-01-14	final	37	7.6	4.33						289	7.266	0.85	8.19E+08	2.19E+07	4.38E+07	9.39E+06	1.60E+07	1.21E+07	2.18E+07	1.27E+08
Barrier	CB1-	2019-01-14	final	47	7.7	4.85						192	7.188	0.898	1.42E+09	2.42E+07	2.40E+07	1.83E+07	1.91E+07	1.62E+07	3.76E+07	2.71E+08
Barrier	CB1	2019-01-14	final	Top sand	7.1	0.67						137	6.039	0.792	1.91E+08	3.55E+05	1.03E+07	4.47E+06	3.66E+06	2.98E+06	3.26E+06	1.74E+07
Barrier	CB1	2019-01-14	final	27	7.0	5.06						173	7.166	0.897	7.38E+08	1.28E+07	3.49E+07	8.18E+06	1.20E+07	9.18E+06	1.52E+07	1.01E+08
Barrier	CB1	2019-01-14	final	37	7.0	4.34						205	7.159	0.873	1.39E+09	3.21E+07	3.25E+07	1.14E+07	2.71E+07	1.22E+07	5.42E+07	1.90E+08
Barrier	CB1	2019-01-14	final	47	7.2	4.77						153	7.076	0.911	4.91E+08	1.40E+07	1.19E+07	3.54E+06	8.34E+06	4.70E+06	1.30E+07	8.27E+07
Barrier	CB2	2019-01-14	final	Top sand	7.0	1.04						178	6.38	0.801	2.28E+08	1.05E+06	2.11E+07	7.18E+06	5.69E+06	7.28E+06	4.73E+06	4.37E+07
Barrier	CB2	2019-01-14	final	27	7.0	5.31						221	7.285	0.876	1.08E+09	1.64E+07	5.29E+07	1.32E+07	2.19E+07	1.10E+07	2.44E+07	2.10E+08
Barrier	CB2	2019-01-14	final	37	7.2	3.66						152	7.059	0.908	5.63E+08	1.35E+07	3.34E+07	6.62E+06	1.24E+07	4.60E+06	1.46E+07	7.26E+07
Barrier	CB2	2019-01-14	final	47	7.4	3.89						218	7.072	0.856	1.23E+09	2.02E+07	2.69E+07	6.74E+06	1.65E+07	8.30E+06	3.18E+07	1.97E+08
Barrier	WCB	2019-01-14	final	Top sand	7.1	0.64						169	6.333	0.798	2.00E+08	1.16E+06	7.19E+06	5.82E+06	4.28E+06	3.38E+06	4.72E+06	2.41E+07
Barrier	WCB	2019-01-14	final	27	7.2	0.69						114	5.586	0.76	1.41E+08	9.96E+05	3.98E+06	2.87E+06	2.98E+06	3.20E+06	2.55E+06	1.10E+07
Barrier	WCB	2019-01-14	final	37	7.2	4.40						96	6.012	0.844	4.75E+08	6.72E+05	4.68E+06	3.16E+06	2.58E+07	7.92E+06	1.40E+07	5.98E+07
Barrier	WCB	2019-01-14	final	47	7.2	8.10						224	7.156	0.86	2.28E+08	5.93E+04	1.84E+06	7.64E+05	1.41E+07	2.97E+06	7.07E+06	2.64E+07
Barrier	Ref	2019-01-14	final	Top sand	7.0	10.7						122	5.273	0.713	2.85E+08	1.84E+05	3.57E+07	3.80E+06	7.57E+06	1.06E+07	3.26E+06	4.89E+07
Barrier	Ref	2019-01-14	final	27	7.3	0.46						137	4.859	0.64	8.48E+07	6.06E+04	5.59E+06	2.66E+06	1.73E+06	5.35E+06	1.20E+06	8.22E+06

Table S2. Primers and reaction conditions for qPCR.

**Gene**<br>Primer ref	Primer pair<br>Forw/Rev	Primer sequence<br>5´- 3´	Final conc<br>(µM)	Melting<br>T (°C)	 t (s)	Annealing<br>T (°C)	 t (s)	Elongation<br>T (°C)	 t (s)	Data acquisition<br>T (°C)	 t (s)	Cycles[a]
nirS	Cd3a Fm	AACGYSAAGGARACSGG	0.50	95	15	65-60[b]	30	72	35	80	5	40
(Throbäck et al., 2004)	R3cdm	GASTTCGGRTGSGTCTTSAYGAA	0.50									
nirK	876F	ATCATGGTSCTGCCGCG	0.50	95	15	63-58[b]	30	72	30	80	5	40
(Henry et al., 2004)	1040 R	GCCTCGATCAGRTTRTGGTT	0.50									
***nosZ*I**	1840 F	CGCRACGGCAASAAGGTSMSSGT	0.50	95	15	65-60[b]	30	72	35	80	5	40
(Henry et al., 2006)	2090 R	CAKRTGCAKSGCRTGGCAGAA	0.50									
***nosZ*II**	nosZII F	CTIGGICCIYTKCAYAC	2.00	95	15	54	30	72	45	80	5	40
(Jones et al., 2013)	nosZII R	GCIGARCARAAITCBGTRC	2.00									
nfrA	nrfAF2aw	CARTGYCAYGTBGARTA	0.50	95	15	57-52[b]	30	72	30	80	5	40
(Welch et al., 2014/Mohan et al., 2004)	nrfAR1	TWNGGCATRTGRCARTC	0.50									
amoA (AOA)	crenamoA 23F	ATGGTCTGGCTWAGACG	0.50	95	15	55	30	72	45	77	5	40
(Tourna et al., 2008)	crenamoA 616R	GCCATCCATCTGTATGTCCA	0.50									
amoA (AOB)	amoA 1F	GGGGTTTCTACTGGTGGT	0.50	95	15	55	30	72	45	77	5	40

amoA (AOB)	amoA 1F	GGGGTTTCTACTGGTGGT	0.50	95	15	55	30	72	45	77	5	40
(Rotthauwe et al., 1997)	amoA 2R	CCCCTCKGSAAAGCCTTCTTC	0.50									

a - Protocols start with an activation step, 95 °C for 5 minutes and are finished with a melt curve, 15 s at 95 °C followed by 65-95 °C in 0.5 °C increments for 5 s.
b - 1 °C decrease per cycle the first six cycles.

References

Henry, S., Baudoin, E., Lopez-Gutierrez, J.C., Martin-Laurent, F., Brauman, A., Philippot, L. 2004. Quantification of denitrifying bacteria in soils by nirK gene targeted real-time PCR. J. Microbiol. Methods 59, 327-335.

Henry, S., Bru, D., Stres, B., Hallet, S., Philippot, L., 2006. Quantitative detection of the nosZ gene, encoding nitrous oxide reductase, and comparison of the abundances of 16S rRNA, narG, nirK, and nosZ genes in soils. Appl. Environ. Microbiol. 72, 5181–5189.

Jones, C., Graf, D., Bru, D., Philippot, L., Hallin, S. 2013. The unaccounted yet abundant nitrous oxide-reducing microbial community: a potential nitrous oxide sink. ISME J. 7, 417-426.

Mohan, S.B., Schmid, M., Jetten, M., Cole, J., 2004. Detection and widespread distribution of the nrfA gene encoding nitrite reduction to ammonia, a short circuit in the biological nitrogen cycle that competes with denitrification. FEMS Microbiol. Ecol. 49, 433–443.

Muyzer, G., Dewaal, E.C., Uitterlinden, A. G. 1993. Profiling of Complex Microbial Populations by Denaturing Gradient Gel-Electroforesis Analysis of Polymerase Chain Reaction-Aplified Genes Coding for 16S Ribosomal RNA. Appl. Environ. Microbiol. 59, 695-700.

Rotthauwe, J.H., Witzel, K.P., Liesack, W. 1997. The ammonia monooxygenase structural gene amoA as a functional marker: molecular fine-scale analysis of natural ammonia-oxidizing populations. Appl. Environ. Microbio.l 63, 4704–4712.

Throbäck, I.N., Enwall, K., Jarvis, A., Hallin, S. 2004. Reassessing PCR primers targeting nirS, nirK and nosZ genes for community surveys of denitrifying bacteria with DGGE. FEMS Microbiol. Ecol. 49, 401-417.

Tourna, M., Freitag, T.E., Nicol, G.W., Prosser, J. I. 2008. Growth, activity and temperature responses of ammonia-oxidizing archaea and bacteria in soil microcosms. Environ. Microbiol. 10, 1357–1364.

Welsh, A., Chee-Sanford, J.C., Connor, L.M., Löffler, F.E., Sanford, R.A. 2014. Refined NrfA Phylogeny Improves PCR-Based nrfA Gene Detection. Appl. Environ. Microbiol. 80, 2110-2119.

Table S3. Correlations between Non-Metric Multidimensional Scaling based on Bray-Curtis distances of the frequent communities in the barrier samples and class level taxonomy, chemical and gene abundance data. Coordinates refer to main Figure 3A. Results from vegan function envfit.

	Variable	Coordinates		Strength	Significance[a]
		MDS1	MDS2	r^2	
Class	Nitrososphaeria	-1.000	0.016	0.189	*
	Methanobacteria	-0.919	0.395	0.037	
	Parcubacteria	0.060	0.998	0.094	
	Fimbriimonadia	0.637	-0.771	0.364	**
	Microgenomatia	0.000	1.000	0.017	
	Bacteroidia	0.823	-0.567	0.222	*
	Saccharimonadia	0.335	-0.942	0.326	**
	Spirochaetia	0.182	-0.983	0.429	**
	Lineage Iia[b]	0.345	0.939	0.208	*
	Blastocatellia	-0.289	-0.957	0.071	
	Thermoleophilia	0.394	-0.919	0.300	**
	Actinobacteria	0.562	-0.827	0.397	**
	Acidimicrobiia	-0.986	-0.165	0.841	**
	Rubrobacteria	-1.000	0.028	0.688	**
	Coriobacteriia	-0.706	0.708	0.206	*
	MB-A2-108	0.663	-0.749	0.141	.
	AKAU4049	0.887	0.462	0.050	
	Desulfitobacteriia	0.592	0.806	0.051	
	Clostridia	0.771	0.636	0.424	**
	Halanaerobiia	-0.993	-0.115	0.125	
	Limnochordia	-0.994	0.113	0.232	*
	Bacilli	-0.972	0.237	0.301	**
	Gitt-GS-136	-0.966	0.258	0.163	.
	KD4-96	-0.964	0.268	0.249	**
	P2-11E	0.795	-0.607	0.253	**
	Anaerolineae	-0.933	0.359	0.723	**
	Chloroflexia	-0.690	0.724	0.608	**
	Dehalococcoidia	-0.998	0.064	0.784	**
	OLB14	-0.735	0.678	0.399	**
	JG30-KF-CM66	-0.699	0.715	0.297	**
	TK10	0.912	-0.411	0.337	**
	Cyanobacteriia	0.800	-0.601	0.031	
	Nitrospira	0.346	0.938	0.460	**
	Fusobacteriia	0.037	0.999	0.132	
	S0134 terrestrial group	-0.865	0.502	0.531	**
	BD2-11terrestrial group	-0.927	0.375	0.692	**
	Longimicrobia	-0.711	-0.703	0.073	
	Gemmatimonadetes	0.623	0.782	0.268	*
	Vampirivibrionia	0.568	-0.823	0.228	*
	Sericytochromatia	0.761	0.649	0.224	*
	Dadabacteriia	0.336	-0.942	0.070	
	Bdellovibrionia	0.915	0.403	0.477	**
	bacteriap25[c]	-0.996	0.085	0.836	**
	uncultured[d]	-0.990	0.141	0.175	*
	Myxococcia	-0.844	0.536	0.137	.
	Desulfobacteria	-0.034	0.999	0.143	.
	Desulfobulbia	-0.797	0.603	0.291	**

	Desulfuromonadia	-0.541	0.841	0.425	**
	Polyangia	-0.301	-0.954	0.311	**
	Oligoflexia	0.985	-0.171	0.297	**
	Alphaproteobacteria	-0.302	-0.953	0.700	**
	Hydrogenedentia	0.682	0.731	0.489	**
	Gammaproteobacteria	0.376	0.927	0.738	**
	Desulfovibrionia	0.168	0.986	0.009	
	Planctomycetes	0.794	-0.608	0.152	.
	Phycisphaerae	-0.382	0.924	0.040	
	Sumerlaeia	0.131	0.991	0.089	
	Verrucomicrobiae	0.641	-0.768	0.439	**
	Subgroup 11[e]	0.816	-0.578	0.194	*
	Subgroup 5[e]	-0.990	0.140	0.379	**
	Acidobacteriae	0.509	0.861	0.102	
	Kapabacteria	0.999	0.038	0.349	**
	SJA-28	0.189	0.982	0.256	*
	Rhodothermia	-0.946	-0.325	0.177	.
	Ignavibacteria	0.244	0.970	0.504	**
	Kryptonia	-0.983	0.184	0.399	**
	Calditrichia	-0.981	0.196	0.271	*
	Methylomirabilia	-0.948	-0.319	0.339	**
	Vicinamibacteria	-0.841	-0.542	0.452	**
	Subgroup 25[e]	-1.000	-0.003	0.607	**
	Subgroup 22[e]	0.716	-0.698	0.073	
	Thermoanaerobaculia	-0.991	0.136	0.397	**
	Holophagae	0.306	0.952	0.373	**
	Entotheonellia	-1.000	-0.017	0.701	**
	Babeliae	0.133	-0.991	0.073	
	Campylobacteria	-0.181	0.983	0.162	.
	Gracilibacteria	-0.613	0.790	0.110	
Chemical	OrgC	-0.699	-0.715	0.209	*
	pH	0.189	-0.982	0.243	*
Gene abundance	16S rRNA	-0.996	0.089	0.559	**
	nrfA	-0.976	0.218	0.493	**
	Archaeal *amoA*	-0.992	0.127	0.578	**
	Bacterial *amoA*	-0.656	0.754	0.265	**
	nirS	-0.754	0.656	0.416	**
	nirK	-0.964	-0.266	0.519	**
	nosZI	-0.861	0.509	0.483	**
	*nosZ*II	-1.000	0.006	0.506	**

[a] p-values adjusted for multiple comparisons using false discovery rate. · p < 0.1, * p < 0.05, ** p < 0.01

[b] Phylum Elusimicrobiota

[c] Phylum Myxococcota

[d] Phylum Desulfobacterota

[e] Phylum Acidobacteriota
